# Editorial: Unlocking crop potential: root characterization through phenomics and genomics

**DOI:** 10.3389/fpls.2026.1838747

**Published:** 2026-04-13

**Authors:** Muhammad Awais Farooq, Gwendolyn K. Kirschner

**Affiliations:** 1 School of Biological Sciences, University of Aberdeen, Aberdeenshire, United Kingdom; 2 Ecological Sciences, The James Hutton Institute, Dundee, United Kingdom

**Keywords:** genomics, hidden half, metabolomics, phenomics, root characterization

## Introduction

Roots lie at the very foundation of plant health, productivity, and resilience. While they remain hidden below the soil surface, their influence on water, nutrient uptake, and interactions with the environment is immense. In a time of climate uncertainty and resource constraints, understanding roots through both phenomics and genomics perspectives offers a pathway to enhance crop adaptation and sustainability. The integration of phenomics (the comprehensive quantification of phenotype at multiple spatial and temporal scales) with genomics (the dissection of genetic architecture and regulatory networks) forms a keystone in modern crop biology. This Research Topic, “Unlocking Crop Potential: Root Characterization through Phenomics and Genomics,” brings together diverse insights into cellular-level processes to field-level performance, highlighting the synergy between advanced phenotyping and genomic tools.

## Multiscale phenomics stress the importance of the root-environment interface

Phenomics allows precise quantification of root responses to environmental variations. At the cellular level, Kaduchová et al. used confocal microscopy to investigate how heat stress affects barley root apical meristems, showing that elevated temperatures above 30 °C disrupt DNA replication and halt mitosis. Their work demonstrates that phenomics can capture the threshold between normal development and stress-induced inhibition on cellular level, providing avenues to breed thermotolerant root systems.

Scaling up from the cellular to organ level, the work of Connolly et al. analyzed maize landraces regarding their growth of aerial roots, and rate of atmospheric N_2_ fixation. Aerial roots in maize secrete a carbohydrate-rich mucilage, which presents a favorable environment for nitrogenase activity by specialized bacteria, enabling the fixation of nitrogen (N) from the atmosphere. They found that up to 36% of plant N was derived from the atmosphere, suggesting that the plant-microbe interaction at the aerial roots could be used to decrease the dependence of maize on synthetic fertilizer. Complementing this, Schuster et al.applied phenotypic screening of tropical maize inbred lines to identify genotypes tolerant to low-phosphorus conditions. By combining shoot and root traits at the seedling stage, they laid the groundwork for selection strategies targeting resource-efficient maize varieties. Their work provides breeders with a selection of inbred lines possessing superior root and shoot traits, demonstrating how direct phenotyping remains foundational for genetic improvement.

## Genetic dissection of root architecture

Advances in genomics and transcriptomics enable the precise mapping of loci, genes, and pathways determining root functionality. In a comprehensive review, Zulfiqar et al. extend our current understanding of the molecular regulatory networks controlling phosphorus utilization efficiency (PUE), emphasizing transcriptional control, post-translational modification, and root–microbe interactions. Their summary of P signaling cascades—linking phosphate transporters, noncoding regulatory elements, and rhizospheric symbioses—provides a framework for translating gene-level discoveries into quantifiable effects on nutrient uptake. Providing a specific example of such a molecular pathway, Yao et al.used transcriptomic analysis to uncover a novel link between nitrate signaling and ethylene biosynthesis in maize roots. By identifying key *ACO* and *ACS* genes, they reveal a regulatory node that could be manipulated to optimize root growth in response to nitrogen availability. Linking such genomic data to spatially resolved root architecture traits can facilitate predictive modeling of root responses under fluctuating nutrient regimes.

The interplay between conserved transcription factor families and developmental regulation is further exemplified by Wang et al., who identified *PgGRAS48* as a candidate root development regulator within the GRAS gene family of *Panax ginseng*. This genome-wide analysis provides new insights into how transcriptional hierarchies, originally characterized in model species, have diversified to govern root morphology in medicinal crops. These findings also broaden the comparative genomic framework necessary for functional annotation across phylogenetically distinct root types.

## From functional genomics to multi-omics integration

Integrative multi-omics approaches further enrich our understanding of root trait diversity. Yang et al.combined metabolomic and transcriptomic profiling in *Tetrastigma hemsleyanum* to identify *ThMYB6* as the regulator of flavonoid pathway genes determining tuberous root coloration. This integrative approach connects secondary metabolism with visible root phenotypes, reflecting how gene regulatory networks translate into phenotypic and ecological adaptations.

At the population level, genome-wide association studies (GWAS) and advanced mapping populations provide powerful tools for linking genotype to phenotype. Chandana et al. identify novel SNPs associated with biological nitrogen fixation traits in chickpea, enabling marker-assisted selection for improved symbiotic efficiency. Similarly, Roy et al. use a multi-parent advanced generation inter-cross (MAGIC) population of 250 rice genotypes to identify root traits and genes associated with methane emissions under flooded conditions, which could facilitate breeding of rice varieties with high yield but low methane emissions. This research advances climate-smart breeding strategies that reduce greenhouse gas emissions without compromising yield.

## Conclusion

Across species and different experimental approaches, three interlinked themes emerge. First, vertical integration across biological scales: phenomics spans from cellular dynamics to whole-plant performance, while genomics resolves variation from SNPs to regulatory networks, bridging the gap from allelic diversity to ecosystem-relevant phenotypes. Second, functional interplay of morphology and metabolism: architectural traits such as root proliferation, nodulation, and aerial root formation are directly linked to nutrient acquisition, nitrogen fixation, and specialized metabolism, revealing root efficiency as a co-optimized trait of structure and biochemical activity. Third, conserved regulatory pathways with translational potential: GRAS, MYB, and ethylene biosynthesis genes appear across diverse species, while species-specific loci reveal how these pathways can be strategically exploited for crop improvement.

## Future perspectives

Integrating high throughput phenomics with multi-omics and microbiome profiling will be essential to move from descriptive associations to predictive models of root function. Machine-learning approaches that connect genomic variation to root traits, combined with standardized phenotyping platforms and shared trait frameworks, can accelerate this process. Incorporating rhizosphere microbial interactions will further capture the full complexity of belowground nutrient cycling. By making the “hidden half” of plants both experimentally accessible and computationally tractable, these strategies open the way to rationally design root systems for improved nutrient-use efficiency, reduced input requirements, and greater resilience. Ultimately, this integrated framework positions roots as dynamic systems whose phenotypic plasticity and genetic regulation can be harnessed to enhance crop productivity, environmental sustainability, and global food security.

